# Supplementary material for: Machine learning application for predicting smoking cessation among US adults: An analysis of waves 1-3 of the PATH study
Source: PLoS One. 2023 Jun 8;18(6):e0286883. doi: 10.1371/journal.pone.0286883 (PMC10249849; doi:10.1371/journal.pone.0286883)
Supplement: S1 Appendix — (PDF) [file pone.0286883.s001.pdf]

S1 Appendix

Steps of data cleaning

Wave 1 PATH survey comprises of 1,742 data columns in total, from variables related to participants' tobacco products history of use and use habits (cigarette, e-cigarette, cigarillo, cigar, filtered cigar, pipe, hookah, smokeless tobacco, snus pouches, and dissolvable tobacco), to demographics and socio-economic status (SES) variables. The Center for the Assessment of Tobacco Regulations (CASTOR) Data Analysis and Dissemination (DAD) Core, [1] provides additional driven variables, such as past 30 days of tobacco use and nicotine dependence score of participants, based on the original survey variables of the PATH study. Tobacco products past 30 days use have previously been considered significant features in cigarette smoking cessation prediction. [2] Therefore, 14 variables on past 30 days use of any single tobacco product, poly tobacco products, combustible tobacco products, and dual use of cigarettes and e-cigarettes in wave 1, and 9 variables on past 30 days use of single, poly, and combustible tobacco products other than cigarettes in wave 2, were provided by the DAD core and added to the original dataset. The nicotine dependence score of individuals in wave 1 calculated by the DAD core was also added to the original dataset. We consider past 30 days use in wave 1 to see if there is an association between participants' tobacco product use in wave 1 and quitting over time in wave 2. Past 30 days use of tobacco products other than cigarettes is also considered for wave 2 to see if the current use of other tobacco products is associated with participants' quitting in wave 2.

The initial dataset, therefore, included 1,766 variables. As the first step of data cleaning, we removed variables that contradicted the baseline definition; in other words, we omitted questions that only targeted never or former smokers. We also removed survey design variables (e.g., weights, variance, and random questions). Any categorical variable with more than 50 levels was removed since features with too many levels overcomplicate the analysis, and many algorithms cannot handle features with more than 50 levels. Variables with no variations (single-level categorical variables) were also removed. Six hundred and seventy variables were removed in the first screening process. We then divided all remaining features into categories as each tobacco product's history of use, use habits, the decision about switching to other tobacco products and/or using other products as additions, past quit attempts, exposure to tobacco advertisements and promotions, noticing tobacco products health warnings, tobacco use habits among family and friends, and rules about tobacco use at home, alcohol and other substances use, history of health problems and medications use, mental and physical health and quality of life, demographics, SES, perceptions and beliefs, and social media exposure. There were multiple similar variables in each category. For our analysis, we selected those variables most relevant to current smokers, which did not target a specific population with the least number of missing samples. Variables were also merged as needed. For instance, in the wave 1 PATH survey, two separate variables show how long a person has smoked fairly regularly in months and years. These two variables were added together and merged into a single "duration of smoking" variable. The dataset at this point included 221 variables.

In the next step, we developed a correlation matrix to diagnose and remove highly correlated variables. Using Pearson's correlation coefficient for numeric variables and Spearman's correlation coefficient for categorical variables, [3] 40 correlated variables (absolute correlation>0.65) [4] were removed to avoid multi-collinearity. The remaining 181 variables (23 numeric features and 158 categorical features) all included questions that targeted either current smokers or all participants; however, some variables included missing samples because participants left the question unanswered. Since we already had a very small sample of quits (710 samples among 9,281), we did not want to

omit any sample due to incomplete answers to some questions. Therefore, instead of simply omitting NAs, we added “missing” as a factor level for the categorical features and labeled NA samples as ”missing” to be able to use them in the analysis. For the few numeric features with NAs, we filled in the NAs with the average value of the variable.

References

1. The Center for the Assessment of Tobacco Regulations Data Analysis and Dissemination Core. Available from: `https://tcors.umich.edu/CoresData.php`.

2. Lai CC, Huang WH, Chang BCC, Hwang LC. Development of Machine Learning Models for Prediction of Smoking Cessation Outcome. International journal of environmental research and public health. 2021;18(5):2584.

3. Jed Wing AWCKAETCZM Steve Weston. find Correlation: Determine highly correlated variables. caret: Classification and Regression Training R package version 60-35. 2014;.

4. Mukaka MM. A guide to appropriate use of correlation coefficient in medical research. Malawi medical journal. 2012;24(3):69–71.
