# Supplementary material for: Machine learning application for predicting smoking cessation among US adults: An analysis of waves 1-3 of the PATH study
Source: PLoS One. 2023 Jun 8;18(6):e0286883. doi: 10.1371/journal.pone.0286883 (PMC10249849; doi:10.1371/journal.pone.0286883)
Supplement: S2 Appendix — (PDF) [file pone.0286883.s002.pdf]

S2 Appendix

Model validation for cessation transition between waves 2-3

To validate the model, we used the best performing algorithm (RF with bagging), with a similar combination of variables to predict the smoking cessation of wave 2 adults (ages 18 and above) current established smokers who became former smokers in wave 3. The variables of each wave of the PATH survey are not the same; some variables appear with a different name, the definitions of some variables are different among waves, some variables are removed from newer waves, and some new variables are added to the newer waves. We tried using the same or similar variables as Table 1 to develop the model for waves 2-3 cessation prediction. However, one variable was not available in wave 2, and two other variables had a slightly different definitions in wave 2. Variable 31 of Table 1, "How often have you noticed things that promote tobacco in the past 6 months?" was not available in wave 2. Additionally, instead of variable 15 of Table 1, "How often do you use the internet?" we used a similar variable in wave 2: "Ever go online to access the Internet or to send and receive e-mails?". We also replaced variable 25 of Table 1, "Used a coupon when buying cigarettes in the past 30 days", with a similar variable in wave 2: "In the past 30 days, how often used a coupon when buying cigarettes?". Variables 1, 4, 5, 7, 9, 10, 12-14, 16, 18-24, 26-30, and 32 of Table 1 were available in wave 2. For variables 2, 3, 6, 8, 11, and 17 (past 30 days use variables), similarly, as waves 1-2, we borrowed them from a derived dataset by the DAD core. [1]

We followed a similar manner as waves 1-2 to develop the baseline sample and the response for waves 2-3. For waves 2-3, we considered the current established cigarette smokers in wave 2 who smoked 100 cigarettes or more in their lifetime and reported smoking every day or some days to track the smoking cessation of those participants in wave 3. In total, 28,362 adult respondents were surveyed in PATH wave 2, among which 25,384 individuals also participated in wave 3. Among adults who participated in both waves 2 and 3, 8,591 individuals were current established smokers in wave 2. Like waves 1-2, for waves 2-3, cigarette smoking cessation was considered for those current established cigarette smokers in wave 2 who did not report smoking a cigarette in the past 30 days and self-reported quitting smoking cigarettes in wave 3. We also removed samples with missing or incomplete responses and merged variables as needed (e.g., smoking duration in months and years). We then filled NAs in variables similarly to waves 1-2. Lastly, some variables of wave 2 of the PATH survey (e.g., the Age range when first started smoking cigarettes fairly regularly) only included information for the "New baseline adult respondents." For those variables, we filled in the information for previous respondents from wave 1.

Among 8,591 current established smokers in wave 2, 622 respondents (7%) quitted smoking in wave 3. We divided the total samples into 70% training and 30% testing sets. An RF model with bagging (the best performing model for waves 1-2), with 31 variables as described, was trained to predict the smoking cessation outcome of participants in wave 3. The performance in the test set resulted in sensitivity = 70%, specificity = 69%, balanced accuracy = 70%, and AUC-ROC = 75%.

References

1. The Center for the Assessment of Tobacco Regulations Data Analysis and Dissemination Core. Available from: `https://tcors.umich.edu/CoresData.php`.
